# Antimicrobial resistance patterns and genomic characterization of *Avibacterium paragallinarum* isolates collected in China from 2013 to 2021

**DOI:** 10.1186/s12866-026-05122-4

**Published:** 2026-05-26

**Authors:** Xing-Ping Li, Guiping Li, Wutong Lin, Donghai Li, Ximin Zeng, Meina Xiu, Yuxin Shao, Fan Yang, Fuzhou Xu, Huiling Sun

**Affiliations:** 1 Beijing Key Laboratory for Prevention and Control of Infectious Diseases in Livestock and Poultry, Institute of Animal Husbandry and Veterinary Medicine, Beijing Academy of Agriculture and Forestry Sciences, Beijing, 100097 China; 2 College of Animal Science and Technology, Henan University of Science and Technology, Luoyang, 471023 China; 3 Department of Animal Science, University of Tennessee, 2505 River Drive, Knoxville, Tennessee USA

**Keywords:** *Avibacterium paragallinarum*, Antibiotics, Antimicrobial susceptibility, Drug resistance gene

## Abstract

**Background:**

*Avibacterium paragallinarum* is the causative agent of infectious coryza in chickens and contributes to significant economic losses in poultry production. Antimicrobial therapy is widely used for disease control; however, increasing antimicrobial resistance (AMR) may compromise treatment efficacy. Long-term surveillance data on AMR in *Av. paragallinarum* in China remain limited.

**Results:**

A total of 117 *Av. paragallinarum* isolates were analyzed, including 113 clinical isolates collected from 13 provinces in China between 2013 and 2021 and 4 historical international reference strains. Antimicrobial susceptibility to 16 antibiotics was assessed using the Kirby–Bauer disk diffusion method. High resistance rates were observed for ampicillin, clindamycin, compound sulfamethoxazole, nalidixic acid, and tetracycline. In contrast, erythromycin, azithromycin, ceftiofur, and spectinomycin retained high levels of susceptibility. Compared with historical strains, contemporary isolates exhibited increased resistance to several antimicrobial classes. Whole-genome sequencing of 15 representative isolates identified multiple resistance genes, including determinants associated with aminoglycoside, β-lactam, sulfonamide, and tetracycline resistance. The tigecycline resistance gene *tet*(X6) was detected in one isolate.

**Conclusion:**

This study first detects gene *tet(X6)* in *Av. paragallinarum* isolates. Continuous surveillance of its multidrug resistance and rational antimicrobial use are vital to curb resistance spread in poultry systems.

**Supplementary Information:**

The online version contains supplementary material available at 10.1186/s12866-026-05122-4.

## Introduction

Infectious coryza, caused by Gram-negative *Avibacterium paragallinarum*, is a severe respiratory disease of chickens that engenders huge economic losses for the poultry industry worldwide [1]. The disease has the characteristics of short incubation period, rapid onset and spread [2]. The disease is harmful to both breeding chickens and laying hens, which can hinder the growth of broilers, reduce the meat quality and decrease the egg production rate by 10% ~ 40% [3]. Based on the Page scheme, *Av. paragallinarum* can be classified into three serovars: A, B and C [4]. *Av. paragallinarum* has appeared in multiple countries, such as the United States, Indonesia, India and the United Kingdom. In China, serovars A, C, and B were identified for the first time in 1987, 1995 and 2003, respectively [2, 5, 6]. Among them, serovar C has become the serovar with more morbidity, while serovar A has been the cof *Av. paragallinarum*.

At present, the most effective preventive measure against *Av. paragallinarum* is vaccination [7]. However, vaccination failures always occur as the emergence of new serovars or serovar variants [8, 9]. In addition to vaccination, antibiotics are also commonly used for sick chickens suffering from infectious coryza. The most widely used antibiotics are sulfonamides, including compound sulfamethoxazole [10]. However, due to the frequent use and even abuse of antibiotics, *Av. paragallinarum* has been gradually resistant to some antibiotics in various countries such as Latin American and Mexico [11, 12]. As a result, some antibiotics have lost their inhibitory effect on *Av. paragallinarum*, eventually causing direct economic losses to chicken farms [13]. There are few studies on the mechanism of drug resistance of *Av. paragallinarum*. Hsu et al. has reported a plasmid of pYHM5 that are associated with drug resistance in *Av. paragallinarum*, which is the first and the only plasmid with multi-drug resistance in the bacteria [3]. This plasmid carries *sul2* gene encoding dihydrobutyrate synthase, *mbeCy* gene encoding mobilization protein and a partial ORF, *strA*, *strB* and *apA*1 gene encoding aminoglycoside phosphotransferase [14]. To date, the comprehensive review of the trend of antimicrobial susceptibility of *Av. paragallinarum* in China has been poorly reported.

In this study, we aimed to investigate the antimicrobial susceptibility and the related drug resistance genes of *Av. paragallinarum* mainly from 2013 to 2021 in China, and better understand the antimicrobial resistance of the bacteria to 16 kinds of antibiotics comprehensively. The antimicrobial susceptibility of the bacteria isolates was tested by Kirby-Bauer method (K-B method) and drug resistance genes were measured through next-generation IIIumina Hiseq × 10 platform sequencing. The results will be of great significance to guide the prevention and treatment of infectious coryza in the future.

## Materials and methods

### Bacterial strains and growth conditions

**Fig. 1:**
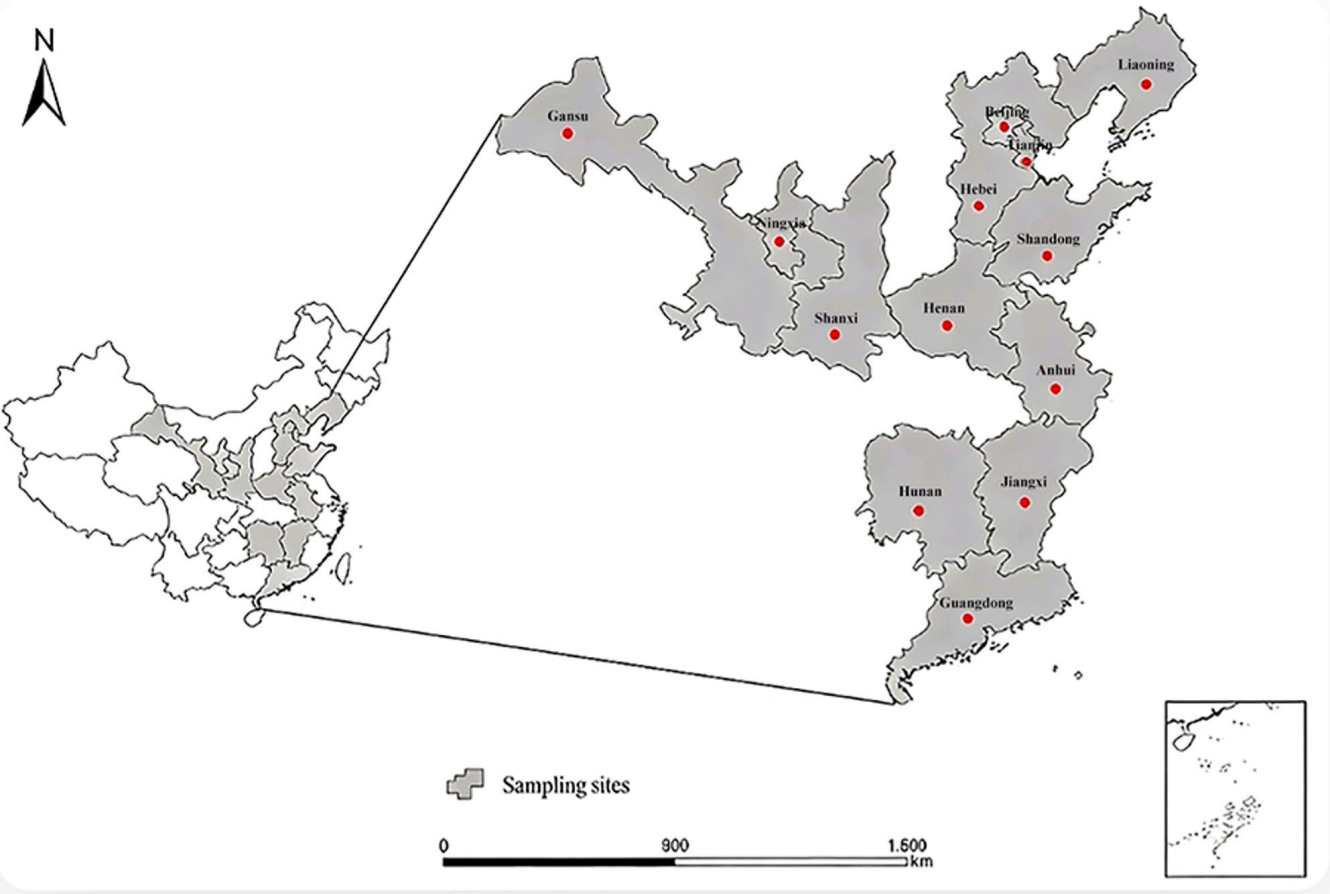
Geographic distribution of *Av. paragallinarum* isolates collected in China (2013–2021). Isolates were obtained from 13 provinces representing major poultry production regions

A total of 117 strains of *Av. paragallinarum* were used in this study, including 113 strains isolated from 2013 to 2021 and 4 international reference isolates from the 20th century. Above 113 clinical strains were isolated from the samples obtained from chicken farms with infectious coryza outbreaks, which were geographically distributed across 13 provinces and municipalities including Beijing, Tianjin, Hebei, Liaoning, Shandong, Henan, Anhui, Jiangxi, Hunan, Guangdong, Shanxi, Gansu and Ningxia (Fig. 1). We obtained informed consent from all animal owners and secured all necessary permits for specimen collection. When performing euthanasia via carbon dioxide (CO₂) inhalation, the initial concentration must be maintained at no less than 40% and promptly elevated to above 70% to minimize animal distress. For the bacteria growth, the strains were inoculated in the tryptic soy broth agar (TSA) plates that were added with 10% chicken serum and 0.0025% nicotinamide adenine dinucleotide (NAD) as previously described [15]. Then, the plates were incubated for 24 h at 37 °C with 5% CO_2_. According to the isolation time, 113 strains from 2013 to 2021 were named as follows:21 − 1 ~ 21 − 16, 20 − 1 ~ 20–22, 19 − 1 ~ 19–29, 18 − 1 ~ 18 − 9, 17 − 1 ~ 17 − 11, 16 − 1 ~ 16 − 8, 15 − 1 ~ 15 − 7, 14 − 1 ~ 14 − 2, 13 − 1 ~ 13 − 9. The four international isolates are strain 221 [16] and 668 [17, 18] isolated from Japan, as well as strain 083 and Modesto [19] isolated from the United States. (Table S1).

### Bacteria identification of *Av. paragallinarum* by PCR

Before antimicrobial susceptibility testing, these 117 isolates of *Av. paragallinarum* were identified by PCR as previously described [20]. Briefly, the colonies of bacterial strains were resuspended in sterile water at 30 µL and boiled for 10 min, and then were placed on ice for 10 min, the supernatant could be used as genomic DNA. For amplification, target template was amplified using the *Av. paragallinarum*-specific primers (forward:5’-TGAGGGTAGTCTTGCACGCGAAT-3′; reverse:5′-CAAGGTATCGATCGTCTCTCTACT-3′). Typically, each reaction contained 10 µL 2 x Taq PCR Mix with loading dye, 1 µL 10 µM of forward primer, 1 µL 10 µM of reverse primer, 2 µL template and 6 µL sterile water. The thermal cycling conditions were as follows: 95 °C 5 min; 95 °C 30 s, 56 °C 30 s, 72 °C 50 s (30 cycle); 72 °C 10 min. The 10 µL final amplification products were validated by 1% agarose gel electrophoresis.

### Antimicrobial susceptibility testing

The antimicrobial susceptibility test was performed by K-B method. The *Av. paragallinarum* strains were inoculated in the trypticase soy broth (TSB) containing 10% chicken serum and 0.0025% NAD, and then were cultured in a shaking incubator at 37℃ until reaching an optical density (OD) of 0.6 at 600 nm (exponential phase of growth). Subsequently, 100 µL bacterial solution was inoculated onto the TSA plate, and antibiotic-impregnated discs were applied. Following incubation at 37 °C under aerobic conditions for 24 h, inhibition zone diameters were measured using electronic calipers and interpreted with reference to Criteria for Antimicrobial Susceptibility Tests (CLSI) as well as the result determination standard of drug susceptibility disk diffusion method provided by Chukiatsiri et al. [13]. In this study, 16 common antibacterial agents against bacterial diseases of livestock and poultry were selected (Table 1).

**Table 1:** The disc contents of 16 antibacterial agents and the result determination standard of drug susceptibility disk diffusion method

Antimicrobials type	Antimicrobials	Disc content (µg/disc)	Inhibition zone diameter (mm)		
			Resistant (*R*)	Intermediary (I)	Susceptible (S)
Penicillins	Ampicillin /AMP	10	≤ 13	14 ~ 16	≥ 17
Lincosamides	Clindamycin /CLN	2	≤ 14	15 ~ 20	≥ 21
Sulfonamides	Compound Sulfamethoxazole /SXT	25	≤ 12	13 ~ 16	≥ 17
Macrolides	Erythromycin /ERM	15	≤ 10	11 ~ 15	≥ 16
	Azithromycin /AZI	15	≤ 8	9 ~ 11	≥ 12
Amphenicols	Florfenicol /FFC	30	≤ 25	26 ~ 28	≥ 29
	Chloramphenicol /CLM	30	≤ 25	26 ~ 28	≥ 29
Quinolones	Enrofloxacin /ENR	5	≤ 16	17 ~ 22	≥ 23
	Nalidixic acid /NAL	30	≤ 13	12 ~ 18	≥ 19
Tetracyclines	Tetracycline /TET	30	≤ 13	14 ~ 22	≥ 23
Cephalosporins	Ceftiofur /FUR	30	≤ 17	18 ~ 20	≥ 21
	Cephalexin /CEX	30	≤ 16	17 ~ 19	≥ 20
Aminoglycosides	Gentamicin /GEM	10	≤ 12	13 ~ 16	≥ 17
	Neomycin /NEO	30	≤ 14	15 ~ 18	≥ 19
	Spectinomycin /SPT	100	≤ 10	11 ~ 13	≥ 14
	Kanamycin /KAN	30	≤ 13	14 ~ 17	≥ 18

### Analysis of resistance genes by next-generation IIIumina Hiseq × 10 platform sequencing

Thirteen *Av. paragallinarum* domestic isolates, spanned a collection period of 9 years and originated from 9 different provinces, representing the natural diversity of the isolate set, along with strain 668 and Modesto were selected for IIIumina sequencing. Cultured bacteria were centrifuged at 14,000 g for 5 min, and the precipitates were sent to Majorbio Bio-Pharm Technology Company (Shanghai, China) for next-generation IIIumina Hiseq × 10 platform sequencing. Resistance genes and plasmid Inc-types were searched by Resfinder (http://genepi.food.dtu.dk/resfinder) and PlasmidFinder (https://cge.food.dtu.dk/services/PlasmidFinder/). Phylogenetic trees for these *Av. paragallinarum* isolates were structured based on the core genome sequences of the isolates using Harvest v1.1.2 [21] with the corresponding characteristics of each isolate visualized using online tool iTOL v4.

### Data processing

All antimicrobial susceptibility data were collected and processed through Origin software. Statistical analyses were performed with R 4.2.0. The Cochran-Armitage trend test was used to assess resistance trends over time. The Mantel-Haenszel test (stratified by year) and a binomial logistic regression model with a year-by-agent interaction term were applied to compare overall resistance rates and temporal trends between two agents.

## Results

### Identification and geographic distribution

The clinical samples were obtained from chicken farms with Infectious coryza outbreaks, which were submitted to our laboratory for post mortem examination. Mucus or caseous material was found in the infraorbital sinus. A total of 113 *Av. paragallinarum* strains were isolated during 2013 to 2021. These isolates were further identified before antimicrobial susceptibility testing. As seen in Fig. 1, the strains were isolated from more than ten intensive chicken farms in 13 provinces and municipalities over a nine-year period, which aimed to make the results of antimicrobial susceptibility of *Av. paragallinarum* in China more convincing. After incubation at 37 ℃ for 24 h in 5% CO_2_ incubator, various gray white colonies were grown on TSA plates that were round, smooth, needle size and dewdrop-like (Fig. S1A). Under the microscope, it could be observed that the colonies were convex and translucent with weak blue fluorescence. However, the bacteria were unable to grow on the common nutrient agar and McConkey agar medium. According to PCR analysis, the specific target fragments at size of 500 bp were amplified for these isolates, which were in line with the expected (Fig. S1B). Therefore, 113 preserved isolates were ensured to be *Av. paragallinarum* again, which could be adopted for the following experiments.

### Antimicrobial susceptibility profiles

Here, the in vitro drug sensitivity tests of 16 antibacterial drugs were carried out. Considering that there were no criteria for determining drug susceptibility results of *Av. paragallinarum* in the CLSI, we tried to develop the related criteria for this kind of bacteria as shown in Table 1. Quantitative distribution of antimicrobial susceptibility of all isolates was determined as summarized in Table 2 and listed in Table S1. Overall, in 2013–2021, more than 80% of *Av. paragallinarum* were susceptible to 4 kinds of antibacterial agents, which included erythromycin (82.1%), azithromycin (99.1%), ceftiofur (97.4%) and spectinomycin (90.6%). Therefore, antimicrobial agents of erythromycin, azithromycin, ceftiofur and spectinomycin had great inhibitory effects on almost all of *Av. paragallinarum*. Nevertheless, there were more than 80% of *Av. paragallinarum* that were resistant to 5 kinds of antibacterial agents, including ampicillin (100.0%), clindamycin (95.7%), compound sulfamethoxazole (83.7%), nalidixic acid (88.9%) and tetracycline (94.9%). These five antimicrobials appear to have low efficacy against most *Av. paragallinarum* isolates and should be used cautiously in clinical practice.

**Table 2:** Quantitative distribution of antimicrobial susceptibility of all *Av. paragallinarum* isolates

Antibacterial agent	Number of isolates (%)		
	S^1^	I^2^	*R* ^3^
AMP	-	-	117 (100%)
CLN	-	4 (4.3%)	113 (95.7%)
SXT	7 (6.0%)	12 (10.3%)	98 (83.7%)
ERM	96 (82.1%)	18 (15.4%)	3 (2.5%)
AZI	116 (99.1%)	-	1 (0.9%)
FFC	80 (68.4%)	30 (25.6%)	7 (6.0%)
CLM	51 (43.6%)	34 (29.1%)	32 (27.3%)
ENR	18 (15.4%)	61 (52.1%)	38 (32.5%)
NAL	9 (7.7%)	4 (3.4%)	104 (88.9%)
TET	-	6 (5.1%)	111 (94.9%)
FUR	114 (97.4%)	1 (0.9%)	2 (1.7%)
CEX	28 (23.9%)	27 (23.1%)	62 (53.0%)
GEM	39 (33.3%)	74 (63.2%)	4 (3.4%)
NEO	6 (5.1%)	53 (45.3%)	58 (49.6%)
SPT	106 (90.6%)	3 (2.6%)	8 (6.8%)
KAN	28 (23.9%)	55 (47.0%)	34 (29.1%)

^1^S=Susceptible. ^2^I= Intermediary. ^3^R=Resistant

**Fig. 2:**
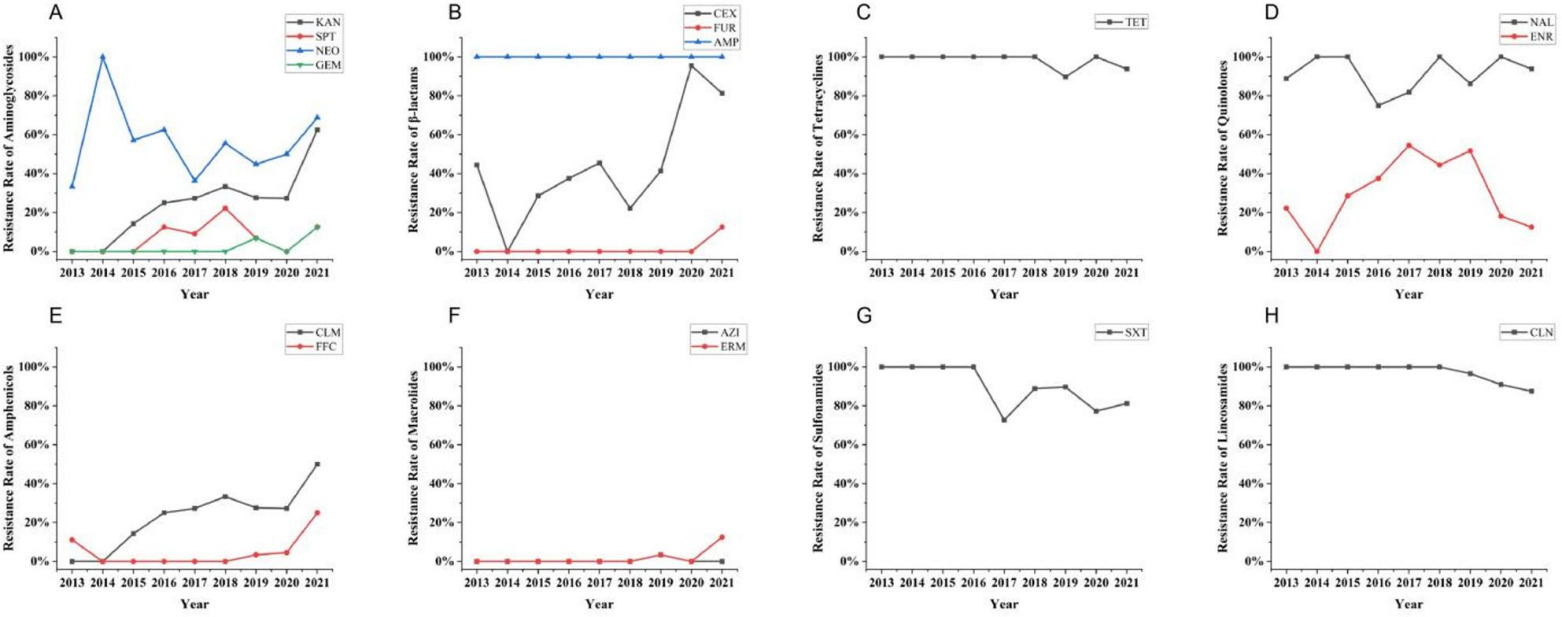
Annual antimicrobial resistance rates of *Av. paragallinarum* isolates from 2013 to 2021. **A**. Aminoglycosides; **B**. β-lactams; **C**. Tetracyclines; **D**. Quinolones; **E**. Amphenicols; **F**. Macrolides; **G**. Sulfonamides; **H**. Lincosamides. Resistance rates (%) to 16 antimicrobial agents were calculated annually using disk diffusion results. Data for 2014 (*n* = 2) should be interpreted with caution due to limited sample size. Abbreviations: AMP, ampicillin; TET, tetracycline; NAL, nalidixic acid; SXT, compound sulfamethoxazole; CEX, cephalexin; ENR, enrofloxacin; ERY, erythromycin; AZM, azithromycin; CEF, ceftiofur; SPC, spectinomycin; etc.Note: The sample size for 2014 (*n*=2) is too small, which may lead to bias in the statistical analysis of annual antimicrobial resistance rates

The temporal trend of the susceptibility of *Av. paragallinarum* to various antimicrobial agents during 2013 to 2021 were illustrated in Fig. 2. The resistance rates of 16 antibiotics were calculated for each year. It should be noted that the resistance rate for 2014 was included despite an insufficient sample size (only two bacterial strains). Compared with the four international strains isolated in the 20th century, the sensitivity to antibiotics such as neomycin, kanamycin, gentamicin, cephalexin, tetracycline, nalidixic acid, enrofloxacin and compound sulfamethoxazole were changed from susceptibility in the 20th century to drug resistance in 2013 (Table S1). Despite temporal variations in antibiotic usage, the susceptibility profiles demonstrated stability across the study period (2013–2021) for the majority of tested agents.

Among aminoglycosides, gentamicin and spectinomycin had a significant inhibitory effect on *Av. paragallinarum* during the surveillance period (Fig. 2A). In 2013 to 2021, the resistance rate of ampicillin was always 100%. Despite that both ceftiofur and cephalexin were cephalosporins, the sensitivity of *Av. paragallinarum* to them varied greatly. Most *Av. paragallinarum* isolates were sensitive to ceftiofur, while were resistant to cephalexin. The resistance rate of cephalexin showed a highly significant increasing trend from 2013 to 2021 ( *P* < 0.001) (Fig. 2B). *Av. paragallinarum* demonstrated sustained resistance to tetracycline, with resistance rates consistently exceeding 90% during 2013 to 2021 (Fig. 2C). For antibiotics of quinolones, the resistance rate of enrofloxacin showed an initial upward trend, followed by a decrease in 2020–2021 (18% and 13%), while *Av. paragallinarum* isolates maintained persistently high resistance levels to nalidixic acid (75.0%-100%) throughout the study period (Fig. 2D). For antibiotics of florfenicol and chloroamphenicol, although both of them were amphenicols, the sensitivity of *Av. paragallinarum* to these two antimicrobials was different significantly (*P* = 0.031) (Fig. 2E). The sensitivity of *Av. paragallinarum* to florfenicol exhibited consistent susceptibility during 2014 to 2018, while developing low-level resistance during 2019 to 2021 with resistance rates rising to 3%-25%. However, the resistance rate to chloramphenicol increased from 0% in 2013 to 50% in 2021. Erythromycin and azithromycin belonged to the macrolides, susceptibility of *Av. paragallinarum* to these two antibiotics remained consistently high throughout the surveillance period, with sensitivity rates of 100% in most years (Fig. 2F). However, the resistance rate of compound sulfamethoxazole and clindamycin remained consistently above 75% and 88%, respectively (Fig. 2G and H).

The resistance profile of *Av. paragallinarum* was also analyzed. A total of 47 resistant patterns were observed in the 117 strains, among which 19 popular patterns were shown in Table 3. The most frequent pattern was CEX-TET-NAL-SXT-CLN-AMP (*n* = 12, 9.9%).

**Table 3:** Antimicrobial resistance patterns identified among isolates

Antimicrobial resistance patterns	Number of AMR classes	Number of strains	Percentage (%)
KAN-NEO-CEX-TET-NAL-ENR-CLM-SXT-CLN-AMP	8	3	2.5%
KAN-NEO-CEX-TET-NAL-CLM-SXT-CLN-AMP	8	5	4.1%
KAN-NEO-TET-NAL-ENR-CLM-SXT-CLN-AMP	7	5	4.1%
KAN-CEX-FUR-TET-NAL-CLM-SXT-CLN-AMP	8	2	1.7%
KAN-SPT-NEO-TET-NAL-CLM-SXT-CLN-AMP	7	2	1.7%
NEO-CEX-TET-NAL-ENR-SXT-CLN-AMP	7	3	2.5%
NEO-CEX-TET-NAL-SXT-CLN-AMP	7	6	5.0%
NEO-TET-NAL-ENR-SXT-CLN-AMP	6	6	5.0%
CEX-TET-NAL-ENR-SXT-CLN-AMP	6	2	1.7%
KAN-TET-NAL-CLM-SXT-CLN-AMP	7	2	1.7%
NEO-TET-NAL-SXT-CLN-AMP	6	6	5.0%
CEX-TET-NAL-SXT-CLN-AMP	6	12	9.9%
TET-NAL-ENR-SXT-CLN-AMP	5	11	9.1%
CEX-TET-NAL-CLN-AMP	5	5	4.1%
NEO-TET-SXT-CLN-AMP	5	3	2.5%
TET-NAL-SXT-CLN-AMP	5	9	7.4%
CEX-TET-CLN-AMP	4	2	1.7%
TET-NAL-CLM-AMP	4	2	1.7%
CLN-AMP	2	2	1.7%

### Genomic characterization of *Av. paragallinarum*

**Fig. 3:**
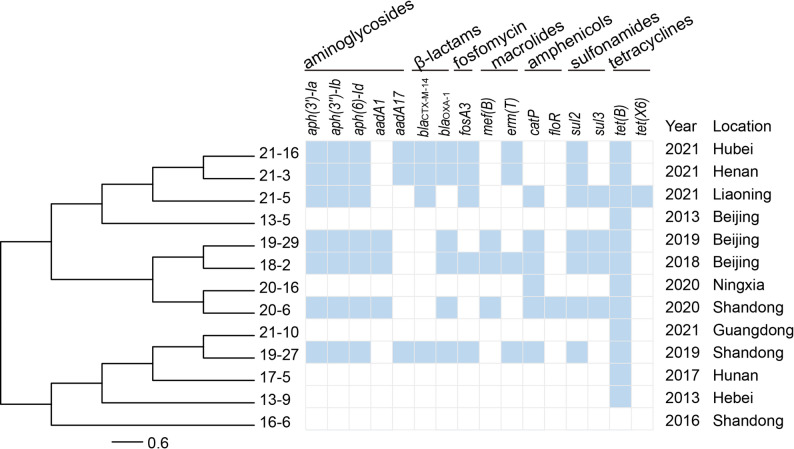
Phylogenetic analysis and resistance genes of the sequenced *Av. paragallinarum* strains. Maximum-likelihood phylogenetic tree constructed from core genome alignment using Harvest. Among the 15 sequenced strains, 13 contemporary isolates (2013–2021) are labeled, with each strain annotated for its resistance gene profile, isolation region, and year. Blank spaces indicate the absence of corresponding resistance genes in the strain. In contrast, two historical strains isolated in the 20th century (668 and Modesto) form a distinct clade, genetically distant from contemporary isolates (2013–2021), and are therefore not shown in this phylogenetic tree

To elucidate the genetic basis of antimicrobial resistance in *Av. paragallinarum*, we randomly selected 13 domestic isolates and 2 historical isolates from our strain collection and conducted whole-genome sequencing using the Illumina platform. The assembled genomes ranged from 2.4 to 2.6 Mb in size. Phylogenetic reconstruction revealed distinct clustering patterns, with historical strains 668 and Modesto (isolated in the 20th century) forming a separate clade distant from contemporary isolates (2013–2021). Due to their divergent placement, these strains were excluded from Fig. 3. This divergence suggests that prevalent strains may have undergone substantial evolutionary changes over time.

The antibiotic resistance genes were analyzed by Resfinder with assembled sequences. Among these strains, the detected resistance genes mainly belonged to the genes resistant to aminoglycosides (*aph(3’)-Ia*, *aph(3’’)-Ib*, *aph(6)-Id*, *aadA1* and *aadA2*), β-lactams (*bla*_CTX−M−14_, *bla*_OXA−1_), fosfomycin (*fosA3)*, macrolides (*mef*(B) and *erm*(T)), amphenicols (*catP* and *floR*), sulfonamides (*sul2* and *sul3*), and tetracyclines (*tet*(B) and *tet*(X6)) (Fig. 3). Among these resistance genes, *tet*(B) was most prevalence (92.3%) (Table 4). As for extended-spectrum β-lactamases, the positive rate of *bla*_OXA−1_ (46.2%) was higher than *bla*_CTX−M−14_ (30.8%). No resistance genes were detected in one isolate from 2016 (16 − 6) and the two historical strains (668 and Modesto). Moreover, none plasmid replicons were identified among all these 15 *Av. paragallinarum* strains via PlasmidFinder, suggesting potential chromosomal localization of resistance genes.

**Table 4:** Drug resistance genes of 13 sequenced *Av. paragallinarum* strains isolates

Antimicrobial class	Resistance gene	No.	Prevalence of resistance genes
Aminoglycosides	*aph(3')-Ia*	7	53.8%
	*aph(3'')-Ib*	7	53.8%
	*aph(6)-Id*	7	53.8%
	*aadA1*	3	23.1%
	*aadA2*	3	23.1%
β-lactams	*bla*_*CTX-M-14*_	4	30.8%
	*bla*_*OXA-1*_	6	46.2%
Fosfomycin	*fosA3*	5	38.5%
Macrolides	*mef(B)*	3	23.1%
	*erm(T)*	4	30.8%
Amphenicols	*catP*	6	46.2%
	*floR*	1	7.7%
Sulfonamides	*sul2*	7	53.8%
	*sul3*	4	30.8%
Tetracyclines	*tet(B)*	12	92.3%
	*tet(X6)*	1	7.7%

The identification of *tet*(X6) among *Av. paragallinarum* isolates represents a particularly concerning finding, as this gene confers resistance to both tetracyclines and the last-resort antibiotic tigecycline. Strain 21 − 5 was further sequenced by Nanopore Sequencing to characterize the ‌tigecycline resistant gene *tet*(X6). The result showed that two copies of *tet*(X6) were located in tRNA-borne genomic islands (GIs) in *Av. paragallinarum* chromosome, accompanied by resistance genes *aph(3’)-Ia*, *aph(3’’)-Ib*, *fosA3*, *bla*_CTX−M−14_, *tet*(B), *catB*, *sul2* and *sul3*.

## Discussion

*Av. paragallinarum*, the pathogen of infectious coryza, is greatly harmful to the chicken industry and has become a research hotspot nowadays. Despite multivalent vaccines have been used in avian farms for a long time, *Av. paragallinarum* infection is still frequently reported worldwide [22]. In this study, we had successively characterized a total of 117 strains of *Av. paragallinarum* from chickens with suspected infectious coryza in the past decade that were collected from 13 provinces and municipalities including Beijing, Hebei, Gansu and Shanxi, etc. Among 117 strains, 113 strains were isolated from 2013 to 2021 and 4 international strains were preserved in the 20th century.

At present, the most common treatment against *Av. paragallinarum* is the use of antibiotics, but it has brought a series of severe drug resistance problems [23, 24]. Although there have been several reports on analysis of antimicrobial susceptibility of *Av. paragallinarum* in China, it remains to be incomprehensive and needs to be further explored [25]. In this study, totally 117 strains of *Av. paragallinarum* were adopted for antimicrobial susceptibility testing. To our knowledge, it is the first time to investigate the susceptibility trend of a variety of *Av. paragallinarum* isolates to related antibacterial agents in China with a large time span.

With implementing a series of significant reforms in veterinary antibiotic use policies since 2020 in China to standardize medication practices and reduce antibiotic misuse in avian industry, resistance rates to enrofloxacin and lincomycin showed a declining trend during the last two years of the observation period (2020–2021), while resistance rates to drugs such as cephalexin still remained at high levels. This may reflect the impact of the veterinary antibiotic policy reforms. However, there is a lag effect in the impact of these policies on antimicrobial resistance of *Av. paragallinarum*. Therefore, the influence of policy implementation still requires continuous monitoring. It’s worth noting that not only in China, this phenomenon also exists in many other countries where infectious coryza outbreaks, such as Dutch and Latin America [13, 26]. In these countries, *Av. paragallinarum* has developed resistant to many kinds of antibacterial agents used for treatment of infectious coryza, such as tetracycline. In this study, though *Av. paragallinarum* was susceptible to the four antimicrobial agents of erythromycin, azithromycin, ceftiofur, and spectinomycin in general, the bacteria had begun to be resistant to them in recent years than 20th century. To be honest, this phenomenon is not optimistic. Importantly, the resistance of *Av. paragallinarum* to antibacterial agents should be paid more attention all over the world.

Based on the next-generation sequencing of 13 selected domestic isolates, it was of great help to understand the related drug resistance mechanism of *Av. paragallinarum*. According to the sequencing data, the presence of multiple drug resistance genes with sequence homology of more than 99% was identified in the genome of the *Av. paragallinarum*. Of which, the *aph(6)-Id*, *aph(3”)-Ib*, *sul2* and *tet*(B) genes have also been reported in *Av. paragallinarum* in recent study [27]. Despite observed 100% phenotypic resistance to ampicillin in *Av. paragallinarum*, the sequences of the extended-spectrum β-lactamases (ESBL) genes were incomplete, which may reflect the undetected β-lactamase variants. Other mechanisms such as insertion elements, transposons, or integron conjugation elements may be also involved in this resistant phenotype [28, 29]. The phenotypic resistance to ampicillin in *Av. paragallinarum* needs to be further investigated.

One of the most notable findings of this study is the identification of the tigecycline resistance gene *tet*(X6) in a contemporary *Av. paragallinarum* isolate. Tet(X) enzymes mediate high-level resistance to tigecycline through flavin-dependent monooxygenase activity, resulting in enzymatic inactivation of tetracycline-class antibiotics [30]. Although tigecycline is not approved for veterinary use, *tet*(X) variants have been increasingly reported in *Enterobacteriaceae* and *Acinetobacter spp*. from animals, food products, and human clinical settings [31–33]. In addition, the *tet*(X) genes can also be present in the same bacteria as *bla*_NDM−1_, resulting in resistance to both tigecycline and carbapenems [34, 35]. The detection of *tet*(X6) in a poultry-associated respiratory pathogen expands the known ecological distribution of this resistance determinant and raises important concerns regarding the potential mobilization and interspecies transfer of last-resort antibiotic resistance genes. The poultry production environment may act as a reservoir for such determinants under selective pressure from tetracycline use. Although plasmid replicons were not identified in this study, further study is required to clarify the potential mobility of *tet*(X6). The *tet*(X6) gene might be transmissible to diverse bacterial hosts—including multiple zoonotic and opportunistic pathogens via GI, integrative and conjugative elements (ICE), plasmids and ISCR2 [30, 35–37]. Its co-localization with other last-resort resistance genes on transferable elements, presents a multifaceted threat that cannot be adequately addressed by poultry surveillance alone [36]. An integrated One Health framework is urgently required to detect, monitor, and mitigate the spread of *tet*(X6) before it becomes widely established.

The multidrug-resistant plasmid could potentially mediate the horizontal transfer of antibiotic resistance genes among bacterial populations. Though a total of 16 kinds of resistance genes have been found, no plasmid Inc-type was identified in the 15 *Av. paragallinarum* isolates in this study. As reported in Hsu’s research, a 5 kb plasmid serves as a mobile genetic element facilitating the horizontal transfer of multiple resistance determinants [3]. We hypothesize that these resistance genes may be either chromosomally encoded or carried by small non-typable plasmids, facilitating their dissemination within this bacterial population.

Although this study encompassed a nine-year surveillance period, several limitations must be acknowledged. Firstly, neither CLSI nor EUCAST provides established breakpoint criteria for *Av. paragallinarum* susceptibility testing. While we have proposed preliminary interpretive criteria (Table 1), these may not accurately reflect true clinical resistance patterns in *Av. paragallinarum* infections. Second, the varying number of annual isolates (particularly the small isolate number of *n* = 2 in 2014) represents another limitation, potentially affecting the robustness of temporal resistance trend analyses and possibly leading to over- or underestimation of resistance rates for certain antimicrobial agents. Additionally, further works need to investigate the consistency between phenotype and genotype of antibiotics resistance in *Av. paragallinarum*. To address these limitations, our future research will adopt a comprehensive collaborative approach to establish species-specific clinical breakpoints through pharmacokinetic/pharmacodynamic (PK/PD) modeling and clinical outcome correlation studies.

## Conclusion

We investigate the antimicrobial susceptibility and resistance genes in 117 strains of *Av. Paragallinarum*, including 113 strains isolated in China from 2013 to 2021 and 4 historical international strains, and illustrate the emerging antimicrobial resistance of the bacteria to 16 kinds of antibiotics comprehensively. Despite temporal variations in antibiotic usage, the susceptibility profiles demonstrated stability across the study period (2013–2021) for the majority of tested agents. Five antimicrobial agents including ampicillin, clindamycin, compound sulfamethoxazole, nalidixic acid and tetracycline have low inhibitory effect on most of *Av. paragallinarum* isolates and should be used with caution in clinical practice. Fortunately, high levels of sensitivity to erythromycin, azithromycin, ceftiofur and spectinomycin are found in China, which can still be used to treat against *Av. paragallinarum* at present. In addition, multiple drug resistance genes, such as *bla*_CTX−M−14_, *fosA3* and *floR*, are detected in *Av. paragallinarum*, and tigecycline resistance gene of *tet*(X6) is also identified in *Av. paragallinarum* for the first time. As *Av. paragallinarum* exhibits consistently high resistance to a broad spectrum of antimicrobial agents, it really requires urgent global attention. At the same time, developments of new prophylactic and therapeutic measures against *Av. paragallinarum* will be an arduous challenge.

## Supplementary Information

Supplementary Material 1.

Supplementary Material 2.

Supplementary Material 3.

Supplementary Material 4.

## Data Availability

The genome sequences of *Av. paragallinarum* strains have been submitted to the GenBank database with the BioProject ID PRJNA1349001 and PRJNA1344445.
