# Supplementary material for: Antimicrobial resistance patterns and genomic characterization of Avibacterium paragallinarum isolates collected in China from 2013 to 2021
Source: BMC Microbiol. 2026 May 26;26:667. doi: 10.1186/s12866-026-05122-4 (PMC13397709; doi:10.1186/s12866-026-05122-4)
Supplement: Supplementary file 1 — Supplementary Material 1. [file 12866_2026_5122_MOESM1_ESM.docx]

**Supporting information**

**Antimicrobial resistance patterns and genomic characterization of *Avibacterium paragallinarum* isolates collected in China from 2013 to 2021**

Xing-Ping Li ^1,2^**^†^**, Guiling Li ^1^ **^†^**, Wutong Lin ^1^**^†^**, Ximin Zeng^3^, Donghai Li ^1^, Meina Xiu ^1^, Yuxin Shao ^1^, Fan Yang ^2^, Fuzhou Xu ^1*^ and Huiling Sun ^1*^

^1^ Beijing Key Laboratory for Prevention and Control of Infectious Diseases in Livestock and Poultry, Institute of Animal Husbandry and Veterinary Medicine, 100097, Beijing Academy of Agriculture and Forestry Sciences, Beijing, China

^2^ College of Animal Science and Technology, Henan University of Science and Technology, Luoyang, 471023, China

^3^ Department of Animal Science, University of Tennessee, Knoxville, TN, United States

**^†^ Contributed to the work equally.**

***** **Corresponding author:**

Dr. Fuzhou Xu and Dr. Huiling Sun, Beijing Key Laboratory for Prevention and Control of Infectious Diseases in Livestock and Poultry, Institute of Animal Husbandry and Veterinary Medicine, Beijing Academy of Agriculture and Forestry Sciences, Beijing, China. Email: xufuzhou@baafs.net.cn; [sunhuiling01@163.com](mailto:sunhuiling01@163.com)

**Table S1. Drug susceptibility determination of *Avibacterium paragallinarum* isolates.**

**Fig. S1 Colony morphology and PCR identification of *Avibacterium paragallinarum* isolates.**

A. Representative colony morphology on TSA supplemented with chicken serum and NAD; B. Agarose gel electrophoresis of PCR amplification products. 1. Positive control; 2. Negative control; 3-17. Isolated strains.
