# Supplementary material for: Antimicrobial resistance patterns and genomic characterization of Avibacterium paragallinarum isolates collected in China from 2013 to 2021
Source: BMC Microbiol. 2026 May 26;26:667. doi: 10.1186/s12866-026-05122-4 (PMC13397709; doi:10.1186/s12866-026-05122-4)
Supplement: Supplementary file 2 — Supplementary Material 2. [file 12866_2026_5122_MOESM2_ESM.docx]

**Table S1**  Drug susceptibility determination of *Avibacterium paragallinarum* isolates.

| Strains | Diameter of bacteriostatic zone (mm)/Judgement result (R/I/S) | | | | | | | | | | | | | | | | | |
| --- | --- | --- | --- | --- | --- | --- | --- | --- | --- | --- | --- | --- | --- | --- | --- | --- | --- | --- |
|  | CS | KAN | SPT | NEO | GEM | CEX | FUR | TGC | TET | NA | ENR | CLM | FFC | AZI | ERM | SXT | CLN | AMP |
| 21-1 | 12/I | 16/I | 18/S | 14/R | 14/I | 17/I | 27/S | 21/R | 11/R | 21/S | 24/S | 28/I | 28/I | 23/S | 15/I | 0/R | 0/R | 0/R |
| 21-2 | 13/I | 0/R | 10/R | 14/R | 17/S | 18/I | 27/S | 22/R | 15/I | 11/R | 19/I | 14/R | 28/I | 23/S | 21/S | 0/R | 12/R | 0/R |
| 21-3 | 6/R | 6/R | 16/S | 6/R | 17/S | 9/R | 24/S | 20/R | 8/R | 6/R | 21/I | 27/I | 28/I | 16/S | 6/R | 6/R | 0/R | 0/R |
| 21-4 | 14/I | 16/I | 23/S | 15/I | 17/S | 7/R | 32/S | 22/R | 9/R | 6/R | 17/I | 31/S | 27/I | 22/S | 17/S | 6/R | 7/R | 0/R |
| 21-5 | 10/R | 0/R | 17/S | 7/R | 12/R | 9/R | 18/I | 20/R | 7/R | 0/R | 21/I | 11/R | 24/R | 20/S | 14/I | 0/R | 0/R | 0/R |
| 21-6 | 11/R | 0/R | 19/S | 9/R | 14/I | 9/R | 15/R | 18/R | 7/R | 0/R | 20/I | 11/R | 28/I | 20/S | 15/I | 0/R | 0/R | 0/R |
| 21-7 | 10/R | 0/R | 18/S | 9/R | 15/I | 9/R | 16/R | 17/R | 6/R | 0/R | 19/I | 10/R | 26/I | 19/S | 14/I | 0/R | 0/R | 0/R |
| 21-8 | 13/I | 0/R | 9/R | 15/I | 20/S | 14/R | 32/S | 23/I | 6/R | 0/R | 21/I | 15/R | 34/S | 27/S | 27/S | 0/R | 17/I | 0/R |
| 21-9 | 14/I | 16/I | 18/S | 13/R | 14/I | 11/R | 21/S | 19/R | 8/R | 8/R | 20/I | 25/R | 25/R | 17/S | 17/S | 9/R | 8/R | 0/R |
| 21-10 | 17/S | 11/R | 19/S | 10/R | 11/R | 12/R | 22/S | 19/R | 9/R | 0/R | 12/R | 27/I | 25/R | 18/S | 18/S | 17/S | 10/R | 0/R |
| 21-11 | 10/R | 21/S | 32/S | 19/S | 19/S | 17/I | 31/S | 25/I | 13/R | 11/R | 24/S | 36/S | 32/S | 27/S | 23/S | 17/S | 20/I | 0/R |
| 21-12 | 12/I | 16/I | 19/S | 14/R | 14/I | 10/R | 23/S | 19/R | 9/R | 8/R | 20/I | 26/I | 25/R | 20/S | 16/S | 12/R | 8/R | 0/R |
| 21-13 | 13/I | 0/R | 15/S | 15/I | 17/S | 13/R | 27/S | 25/I | 13/R | 9/R | 16/R | 14/R | 28/I | 23/S | 21/S | 0/R | 14/R | 0/R |
| 21-14 | 11/R | 0/R | 14/S | 11/R | 14/I | 14/R | 22/S | 20/R | 9/R | 8/R | 19/I | 11/R | 26/I | 20/S | 14/I | 0/R | 0/R | 0/R |
| 21-15 | 12/I | 18/S | 19/S | 18/I | 17/S | 12/R | 31/S | 25/I | 11/R | 9/R | 22/I | 31/S | 32/S | 24/S | 20/S | 14/I | 11/R | 0/R |
| 21-16 | 15/S | 0/R | 23/S | 0/R | 23/S | 13/R | 31/S | 27/I | 9/R | 6/R | 26/S | 30/S | 33/S | 20/S | 0/R | 0/R | 0/R | 0/R |
| 20-1 | 13/I | 0/R | 16/S | 11/R | 18/S | 14/R | 27/S | 22/R | 7/R | 0/R | 10/R | 11/R | 30/S | 22/S | 16/S | 0/R | 0/R | 0/R |
| 20-2 | 12/I | 0/R | 16/S | 13/R | 17/S | 16/R | 26/S | 20/R | 9/R | 7/R | 17/I | 10/R | 26/I | 22/S | 15/I | 0/R | 6/R | 0/R |
| 20-3 | 12/I | 18/S | 20/S | 16/I | 18/S | 16/R | 31/S | 20/R | 12/R | 9/R | 21/I | 31/S | 30/S | 24/S | 21/S | 13/I | 6/R | 0/R |
| 20-4 | 13/I | 0/R | 16/S | 12/R | 18/S | 15/R | 25/S | 24/I | 13/R | 11/R | 16/R | 11/R | 28/I | 24/S | 20/S | 0/R | 14/R | 0/R |
| 20-5 | 13/I | 20/S | 24/S | 17/I | 17/S | 13/R | 26/S | 24/I | 8/R | 0/R | 22/I | 31/S | 30/S | 25/S | 17/S | 6/R | 0/R | 0/R |
| 20-6 | 16/S | 0/R | 20/S | 15/I | 20/S | 14/R | 31/S | 25/I | 12/R | 0/R | 22/I | 12/R | 17/R | 27/S | 16/S | 0/R | 0/R | 0/R |
| 20-7 | 12/I | 14/I | 21/S | 17/I | 15/I | 11/R | 23/S | 19/R | 8/R | 7/R | 21/I | 28/I | 29/S | 20/S | 16/S | 13/I | 10/R | 0/R |
| 20-8 | 13/I | 17/I | 23/S | 17/I | 16/I | 12/R | 25/S | 21/R | 9/R | 7/R | 21/I | 30/S | 28/I | 22/S | 16/S | 14/I | 10/R | 0/R |
| 20-9 | 13/I | 14/I | 23/S | 16/I | 16/I | 13/R | 23/S | 20/R | 9/R | 8/R | 21/I | 30/S | 30/S | 23/S | 18/S | 12/R | 10/R | 0/R |
| 20-10 | 10/R | 16/I | 21/S | 13/R | 15/I | 15/R | 24/S | 23/I | 10/R | 8/R | 21/I | 29/S | 31/S | 23/S | 19/S | 6/R | 10/R | 0/R |
| 20-11 | 10/R | 14/I | 22/S | 13/R | 14/I | 14/R | 23/S | 22/R | 9/R | 8/R | 22/I | 28/I | 30/S | 23/S | 18/S | 6/R | 9/R | 0/R |
| 20-12 | 10/R | 14/I | 21/S | 11/R | 16/I | 13/R | 23/S | 21/R | 9/R | 8/R | 21/I | 27/I | 28/I | 22/S | 17/S | 6/R | 9/R | 0/R |
| 20-13 | 13/I | 0/R | 20/S | 12/R | 15/I | 13/R | 26/S | 23/I | 9/R | 0/R | 10/R | 30/S | 29/S | 23/S | 18/S | 0/R | 6/R | 0/R |
| 20-14 | 11/R | 14/I | 19/S | 13/R | 18/S | 13/R | 22/S | 20/R | 10/R | 7/R | 19/I | 27/I | 29/S | 20/S | 15/I | 6/R | 6/R | 0/R |
| 20-15 | 12/I | 0/R | 13/I | 11/R | 14/I | 13/R | 26/S | 22/R | 9/R | 6/R | 14/R | 12/R | 27/I | 21/S | 15/I | 0/R | 6/R | 0/R |
| 20-16 | 14/I | 19/S | 20/S | 18/I | 19/S | 17/I | 29/S | 25/I | 13/R | 10/R | 24/S | 14/R | 30/S | 25/S | 23/S | 20/S | 15/I | 0/R |
| 20-17 | 13/I | 17/I | 20/S | 16/I | 14/I | 11/R | 26/S | 20/R | 8/R | 0/R | 21/I | 27/I | 30/S | 22/S | 19/S | 0/R | 18/I | 0/R |
| 20-18 | 12/I | 16/I | 22/S | 17/I | 16/I | 13/R | 25/S | 20/R | 8/R | 6/R | 22/I | 32/S | 30/S | 22/S | 18/S | 0/R | 0/R | 0/R |
| 20-19 | 12/I | 18/S | 24/S | 16/I | 18/S | 14/R | 27/S | 23/I | 10/R | 6/R | 22/I | 31/S | 30/S | 26/S | 20/S | 0/R | 8/R | 0/R |
| 20-20 | 11/R | 15/I | 20/S | 13/R | 14/I | 14/R | 23/S | 20/R | 10/R | 10/R | 19/I | 27/I | 28/I | 20/S | 18/S | 13/I | 8/R | 0/R |
| 20-21 | 11/R | 15/I | 21/S | 15/I | 15/I | 16/R | 22/S | 21/R | 9/R | 10/R | 19/I | 28/I | 27/I | 20/S | 16/S | 0/R | 8/R | 0/R |
| 20-22 | 14/I | 17/I | 20/S | 14/R | 15/I | 11/R | 26/S | 23/I | 9/R | 6/R | 19/I | 28/I | 30/S | 22/S | 18/S | 9/R | 9/R | 0/R |
| 19-1 | 12/I | 17/I | 20/S | 15/I | 15/I | 12/R | 22/S | 21/R | 9/R | 8/R | 19/I | 29/S | 27/I | 22/S | 17/S | 7/R | 9/R | 0/R |
| 19-2 | 13/I | 0/R | 18/S | 14/R | 17/S | 13/R | 24/S | 21/R | 10/R | 12/R | 21/I | 13/R | 29/S | 24/S | 18/S | 0/R | 11/R | 0/R |
| 19-3 | 12/I | 0/R | 0/R | 0/R | 0/R | 16/R | 35/S | 27/I | 15/I | 0/R | 0/R | 13/R | 27/I | 0/R | 17/S | 0/R | 0/R | 0/R |
| 19-4 | 14/I | 0/R | 16/S | 15/I | 18/S | 18/I | 28/S | 24/I | 11/R | 10/R | 20/I | 11/R | 29/S | 25/S | 20/S | 0/R | 10/R | 0/R |
| 19-5 | 15/S | 19/S | 24/S | 16/I | 19/S | 17/I | 35/S | 23/I | 13/R | 22/S | 28/S | 35/S | 35/S | 27/S | 21/S | 6/R | 9/R | 0/R |
| 19-6 | 14/I | 6/R | 21/S | 12/R | 18/S | 14/R | 26/S | 24/I | 9/R | 6/R | 8/R | 29/S | 30/S | 23/S | 17/S | 0/R | 6/R | 0/R |
| 19-7 | 14/I | 15/I | 19/S | 13/R | 12/R | 11/R | 27/S | 20/R | 10/R | 18/I | 22/I | 26/I | 27/I | 21/S | 19/S | 0/R | 9/R | 0/R |
| 19-8 | 12/I | 20/S | 24/S | 17/I | 17/S | 13/R | 26/S | 20/R | 11/R | 0/R | 10/R | 28/I | 29/S | 25/S | 17/S | 0/R | 0/R | 0/R |
| 19-9 | 15/S | 17/I | 20/S | 16/I | 14/I | 18/I | 29/S | 23/I | 9/R | 10/R | 22/I | 31/S | 31/S | 24/S | 18/S | 0/R | 0/R | 0/R |
| 19-10 | 14/I | 20/S | 20/S | 18/I | 18/S | 26/S | 30/S | 24/I | 15/I | 10/R | 22/I | 34/S | 32/S | 25/S | 25/S | 13/I | 20/I | 0/R |
| 19-11 | 15/S | 16/I | 21/S | 16/I | 16/I | 26/S | 28/S | 23/I | 10/R | 10/R | 11/R | 31/S | 31/S | 22/S | 19/S | 0/R | 0/R | 0/R |
| 19-12 | 12/I | 17/I | 21/S | 13/R | 16/I | 21/S | 22/S | 22/R | 9/R | 10/R | 22/I | 28/I | 27/I | 21/S | 18/S | 0/R | 0/R | 0/R |
| 19-13 | 13/I | 15/I | 20/S | 13/R | 15/I | 18/I | 22/S | 20/R | 9/R | 9/R | 20/I | 27/I | 28/I | 21/S | 15/S | 0/R | 0/R | 0/R |
| 19-14 | 14/I | 16/I | 22/S | 18/I | 17/S | 14/R | 25/S | 20/R | 9/R | 0/R | 21/I | 31/S | 30/S | 25/S | 21/S | 0/R | 0/R | 0/R |
| 19-15 | 13/I | 18/S | 20/S | 16/I | 14/I | 12/R | 27/S | 22/R | 8/R | 8/R | 21/I | 28/I | 32/S | 24/S | 18/S | 16/I | 0/R | 0/R |
| 19-16 | 17/S | 18/S | 20/S | 15/I | 15/I | 27/S | 33/S | 24/I | 9/R | 10/R | 10/R | 31/S | 33/S | 23/S | 18/S | 0/R | 0/R | 0/R |
| 19-17 | 13/I | 0/R | 10/R | 10/R | 13/I | 17/I | 25/S | 23/I | 10/R | 15/I | 15/R | 12/R | 31/S | 24/S | 14/I | 0/R | 0/R | 0/R |
| 19-18 | 14/I | 0/R | 21/S | 14/R | 17/S | 18/I | 29/S | 22/R | 10/R | 9/R | 13/R | 15/R | 33/S | 26/S | 20/S | 0/R | 9/R | 0/R |
| 19-19 | 15/S | 16/I | 20/S | 15/I | 14/I | 20/S | 25/S | 23/I | 12/R | 12/R | 14/R | 30/S | 27/I | 23/S | 18/S | 0/R | 0/R | 0/R |
| 19-20 | 15/S | 17/I | 20/S | 14/R | 15/I | 20/S | 29/S | 23/I | 10/R | 11/R | 14/R | 30/S | 30/S | 21/S | 19/S | 0/R | 0/R | 0/R |
| 19-21 | 15/S | 20/S | 19/S | 15/I | 14/I | 20/S | 26/S | 23/I | 12/R | 11/R | 14/R | 31/S | 28/I | 25/S | 18/S | 0/R | 0/R | 0/R |
| 19-22 | 15/S | 16/I | 20/S | 16/I | 14/I | 21/S | 27/S | 22/R | 10/R | 11/R | 13/R | 30/S | 30/S | 24/S | 16/S | 0/R | 0/R | 0/R |
| 19-23 | 16/S | 17/I | 24/S | 14/R | 15/I | 18/I | 30/S | 22/R | 10/R | 7/R | 12/R | 30/S | 28/I | 23/S | 18/S | 0/R | 0/R | 0/R |
| 19-24 | 16/S | 16/I | 23/S | 14/R | 16/I | 21/S | 26/S | 22/R | 9/R | 10/R | 15/R | 30/S | 29/S | 25/S | 16/S | 0/R | 0/R | 0/R |
| 19-25 | 15/S | 0/R | 15/S | 14/R | 18/S | 18/I | 32/S | 24/I | 10/R | 8/R | 16/R | 14/R | 35/S | 29/S | 21/S | 0/R | 0/R | 0/R |
| 19-26 | 14/I | 20/S | 24/S | 16/I | 16/I | 12/R | 32/S | 25/I | 11/R | 22/S | 29/S | 32/S | 35/S | 32/S | 24/S | 6/R | 10/R | 0/R |
| 19-27 | 0/R | 0/R | 14/S | 0/R | 15/I | 9/R | 25/S | 25/R | 9/R | 0/R | 11/R | 8/R | 30/S | 18/S | 6/R | 0/R | 0/R | 0/R |
| 19-28 | 14/I | 20/S | 24/S | 18/I | 19/S | 16/R | 30/S | 25/R | 14/I | 11/R | 24/S | 13/R | 29/S | 24/S | 20/S | 16/I | 12/R | 0/R |
| 19-29 | 13/I | 19/S | 21/S | 16/I | 17/S | 11/R | 24/S | 21/R | 9/R | 8/R | 18/I | 28/I | 25/R | 23/S | 19/S | 15/I | 0/R | 0/R |
| 18-1 | 13/I | 17/I | 21/S | 15/I | 16/I | 23/S | 29/S | 25/I | 11/R | 9/R | 15/R | 30/S | 32/S | 27/S | 20/S | 0/R | 0/R | 0/R |
| 18-2 | 14/I | 0/R | 18/S | 14/R | 14/I | 15/R | 30/S | 24/I | 12/R | 9/R | 20/I | 11/R | 35/S | 25/S | 19/S | 0/R | 0/R | 0/R |
| 18-3 | 13/I | 0/R | 0/R | 0/R | 14/I | 23/S | 25/S | 24/I | 9/R | 9/R | 20/I | 12/R | 30/S | 20/S | 15/I | 0/R | 0/R | 0/R |
| 18-4 | 14/I | 16/I | 20/S | 15/I | 16/I | 20/S | 24/S | 22/R | 7/R | 6/R | 18/I | 30/S | 31/S | 26/S | 18/S | 0/R | 0/R | 0/R |
| 18-5 | 13/I | 19/S | 21/S | 18/I | 16/I | 17/I | 29/S | 22/R | 10/R | 11/R | 20/I | 31/S | 32/S | 25/S | 17/S | 14/I | 11/R | 0/R |
| 18-6 | 15/S | 21/S | 25/S | 19/S | 22/S | 12/R | 32/S | 25/I | 12/R | 8/R | 13/R | 35/S | 34/S | 31/S | 23/S | 12/R | 7/R | 0/R |
| 18-7 | 13/I | 0/R | 9/R | 12/R | 14/I | 18/I | 25/S | 21/R | 9/R | 8/R | 18/I | 14/R | 32/S | 23/S | 11/I | 0/R | 0/R | 0/R |
| 18-8 | 17/R | 17/I | 21/S | 14/R | 16/I | 22/S | 30/S | 22/R | 10/R | 11/R | 11/R | 31/S | 30/S | 22/S | 19/S | 0/R | 0/R | 0/R |
| 18-9 | 14/I | 16/I | 21/S | 14/R | 13/I | 19/I | 27/S | 25/I | 11/R | 10/R | 14/R | 31/S | 31/S | 24/S | 17/S | 0/R | 0/R | 0/R |
| 17-1 | 14/I | 16/I | 22/S | 15/I | 15/I | 19/I | 29/S | 24/I | 10/R | 9/R | 23/S | 31/S | 30/S | 22/S | 17/S | 0/R | 0/R | 0/R |
| 17-2 | 13/I | 0/R | 16/S | 14/R | 15/I | 17/I | 25/S | 20/R | 8/R | 0/R | 13/R | 11/R | 31/S | 25/S | 17/S | 0/R | 0/R | 0/R |
| 17-3 | 13/I | 0/R | 11/I | 10/R | 15/I | 19/I | 27/S | 26/I | 12/R | 9/R | 15/R | 15/R | 32/S | 22/S | 14/I | 0/R | 0/R | 0/R |
| 17-4 | 15/S | 19/S | 23/S | 19/S | 17/S | 12/R | 26/S | 21/R | 11/R | 20/S | 29/S | 32/S | 29/S | 28/S | 20/S | 17/S | 12/R | 0/R |
| 17-5 | 15/S | 17/I | 12/I | 15/I | 16/I | 12/R | 23/S | 22/R | 10/R | 18/I | 25/S | 27/I | 29/S | 23/S | 18/S | 14/I | 9/R | 0/R |
| 17-6 | 14/I | 19/S | 23/S | 17/I | 17/S | 13/R | 28/S | 23/I | 9/R | 8/R | 11/R | 31/S | 30/S | 26/S | 20/S | 13/I | 11/R | 0/R |
| 17-7 | 13/I | 17/I | 21/S | 15/I | 15/I | 20/S | 27/S | 24/I | 12/R | 12/R | 16/R | 32/S | 31/S | 24/S | 17/S | 0/R | 0/R | 0/R |
| 17-8 | 13/I | 14/I | 20/S | 14/R | 14/I | 15/R | 27/S | 21/R | 9/R | 8/R | 14/R | 26/I | 28/I | 21/S | 16/S | 0/R | 0/R | 0/R |
| 17-9 | 13/I | 0/R | 0/R | 10/R | 14/I | 15/R | 21/S | 20/R | 9/R | 8/R | 19/I | 11/R | 30/S | 21/S | 12/I | 0/R | 0/R | 0/R |
| 17-10 | 13/I | 18/S | 20/S | 15/I | 16/I | 23/S | 28/S | 23/I | 10/R | 9/R | 15/R | 30/S | 32/S | 22/S | 17/S | 0/R | 0/R | 0/R |
| 17-11 | 16/S | 20/S | 23/S | 17/I | 18/S | 23/S | 32/S | 24/I | 8/R | 10/R | 21/I | 31/S | 32/S | 23/S | 19/S | 0/R | 0/R | 0/R |
| 16-1 | 16/S | 17/I | 20/S | 13/R | 15/I | 23/S | 28/S | 23/I | 10/R | 25/S | 27/S | 28/I | 31/S | 23/S | 17/S | 0/R | 0/R | 0/R |
| 16-2 | 12/I | 0/R | 0/R | 9/R | 13/I | 23/S | 30/S | 20/R | 10/R | 8/R | 23/S | 15/R | 30/S | 22/S | 14/I | 0/R | 0/R | 0/R |
| 16-3 | 16/S | 20/S | 21/S | 18/I | 19/S | 27/S | 29/S | 23/I | 10/R | 10/R | 13/R | 30/S | 33/S | 26/S | 23/S | 0/R | 0/R | 0/R |
| 16-4 | 13/I | 0/R | 20/S | 0/R | 14/I | 14/R | 21/S | 23/I | 9/R | 9/R | 22/I | 0/R | 29/S | 22/S | 17/S | 0/R | 0/R | 0/R |
| 16-5 | 12/I | 14/I | 19/S | 15/I | 14/I | 18/I | 25/S | 21/R | 11/R | 11/R | 15/R | 28/I | 28/I | 22/S | 16/S | 0/R | 0/R | 0/R |
| 16-6 | 13/I | 15/I | 19/S | 14/R | 13/I | 14/R | 29/S | 22/R | 9/R | 19/S | 23/S | 28/I | 30/S | 23/S | 18/S | 8/R | 0/R | 0/R |
| 16-7 | 13/I | 15/I | 19/S | 14/R | 15/I | 16/R | 27/S | 22/R | 9/R | 8/R | 14/R | 28/I | 31/S | 22/S | 17/S | 0/R | 0/R | 0/R |
| 16-8 | 15/S | 16/I | 23/S | 15/I | 15/I | 17/I | 26/S | 21/R | 7/R | 8/R | 17/I | 26/I | 30/S | 24/S | 15/I | 0/R | 0/R | 0/R |
| 15-1 | 15/S | 7/R | 19/S | 13/R | 19/S | 18/I | 24/S | 21/R | 8/R | 8/R | 12/R | 20/R | 30/S | 24/S | 19/S | 0/R | 0/R | 0/R |
| 15-2 | 13/I | 14/I | 20/S | 15/I | 16/I | 17/I | 26/S | 21/R | 9/R | 9/R | 13/R | 30/S | 30/S | 21/S | 14/I | 0/R | 0/R | 0/R |
| 15-3 | 14/I | 18/S | 20/S | 14/R | 16/I | 23/S | 26/S | 22/R | 7/R | 8/R | 20/I | 28/I | 32/S | 23/S | 20/S | 0/R | 0/R | 0/R |
| 15-4 | 13/I | 15/I | 20/S | 14/R | 14/I | 18/I | 26/S | 21/R | 10/R | 9/R | 19/I | 27/I | 28/I | 22/S | 19/S | 0/R | 0/R | 0/R |
| 15-5 | 13/I | 15/I | 19/S | 14/R | 14/I | 16/R | 28/S | 20/R | 0/R | 0/R | 19/I | 28/I | 28/I | 23/S | 17/S | 0/R | 0/R | 0/R |
| 15-6 | 14/I | 15/I | 19/S | 15/I | 15/I | 21/S | 24/S | 21/R | 7/R | 0/R | 19/I | 30/S | 28/I | 22/S | 19/S | 0/R | 0/R | 0/R |
| 15-7 | 14/I | 19/S | 20/S | 17/I | 14/I | 15/R | 21/S | 21/R | 9/R | 0/R | 21/I | 29/S | 31/S | 23/S | 19/S | 0/R | 0/R | 0/R |
| 14-1 | 16/S | 18/S | 20/S | 14/R | 16/I | 20/S | 20/I | 22/R | 9/R | 9/R | 20/I | 28/I | 30/S | 20/S | 15/I | 0/R | 0/R | 0/R |
| 14-2 | 12/I | 15/I | 26/S | 14/R | 14/I | 20/S | 25/S | 22/R | 9/R | 10/R | 18/I | 28/I | 31/S | 23/S | 17/S | 0/R | 0/R | 0/R |
| 13-1 | 14/I | 16/I | 19/S | 14/R | 14/I | 18/I | 27/S | 21/R | 12/R | 14/I | 25/S | 29/S | 29/S | 23/S | 21/S | 0/R | 10/R | 0/R |
| 13-2 | 15/S | 15/I | 19/S | 15/I | 14/I | 22/S | 28/S | 22/R | 9/R | 10/R | 28/S | 30/S | 33/S | 25/S | 18/S | 0/R | 0/R | 0/R |
| 13-3 | 13/I | 15/I | 20/S | 13/R | 13/I | 18/I | 26/S | 20/R | 10/R | 11/R | 11/R | 26/I | 28/I | 21/S | 15/I | 0/R | 0/R | 0/R |
| 13-4 | 15/S | 15/I | 20/S | 15/I | 15/I | 24/S | 25/S | 22/R | 7/R | 6/R | 18/I | 30/S | 30/S | 24/S | 17/S | 0/R | 0/R | 0/R |
| 13-5 | 14/I | 15/I | 20/S | 16/I | 15/I | 15/R | 31/S | 24/I | 8/R | 0/R | 20/I | 27/I | 28/I | 24/S | 16/S | 0/R | 0/R | 0/R |
| 13-6 | 18/S | 17/I | 20/S | 16/I | 15/I | 13/R | 23/S | 20/R | 0/R | 0/R | 18/I | 28/I | 29/S | 25/S | 16/S | 0/R | 0/R | 0/R |
| 13-7 | 14/I | 17/I | 20/S | 17/I | 17/S | 19/I | 28/S | 23/I | 10/R | 8/R | 22/I | 29/S | 29/S | 23/S | 18/S | 0/R | 0/R | 0/R |
| 13-8 | 14/I | 16/I | 19/S | 15/I | 13/I | 15/R | 25/S | 21/R | 10/R | 9/R | 19/I | 29/S | 29/S | 21/S | 17/S | 0/R | 0/R | 0/R |
| 13-9 | 13/I | 15/I | 19/S | 13/R | 15/I | 11/R | 21/S | 20/R | 9/R | 6/R | 16/R | 26/I | 25/R | 21/S | 17/S | 0/R | 7/R | 0/R |
| 221 | 15/S | 0/R | 21/S | 16/I | 18/S | 18/I | 30/S | 26/I | 11/R | 10/R | 21/I | 15/R | 34/S | 26/S | 18/R | 0/R | 11R | 0/R |
| 0083 | 20/S | 26/S | 30/S | 24/S | 28/S | 22/S | 32/S | 24/I | 13/R | 26/S | 30/S | 35/S | 34/S | 28/S | 28/S | 32/S | 12/R | 0/R |
| 668 | 14/I | 23/S | 26/S | 20/S | 21/S | 26/S | 36/S | 30/S | 18/I | 34/S | 35/S | 35/S | 35/S | 29/S | 24/S | 31/S | 7/R | 0/R |
| Modesto | 15/S | 24/S | 22/S | 22/S | 22/S | 22/S | 35/S | 28/I | 18/I | 31/S | 35/S | 35/S | 35/S | 26/S | 24/S | 28/S | 14/R | 0/R |
