# Supplementary figures and images for: Antimicrobial resistance patterns and genomic characterization of Avibacterium paragallinarum isolates collected in China from 2013 to 2021
Source: BMC Microbiol. 2026 May 26;26:667. doi: 10.1186/s12866-026-05122-4 (PMC13397709; doi:10.1186/s12866-026-05122-4)

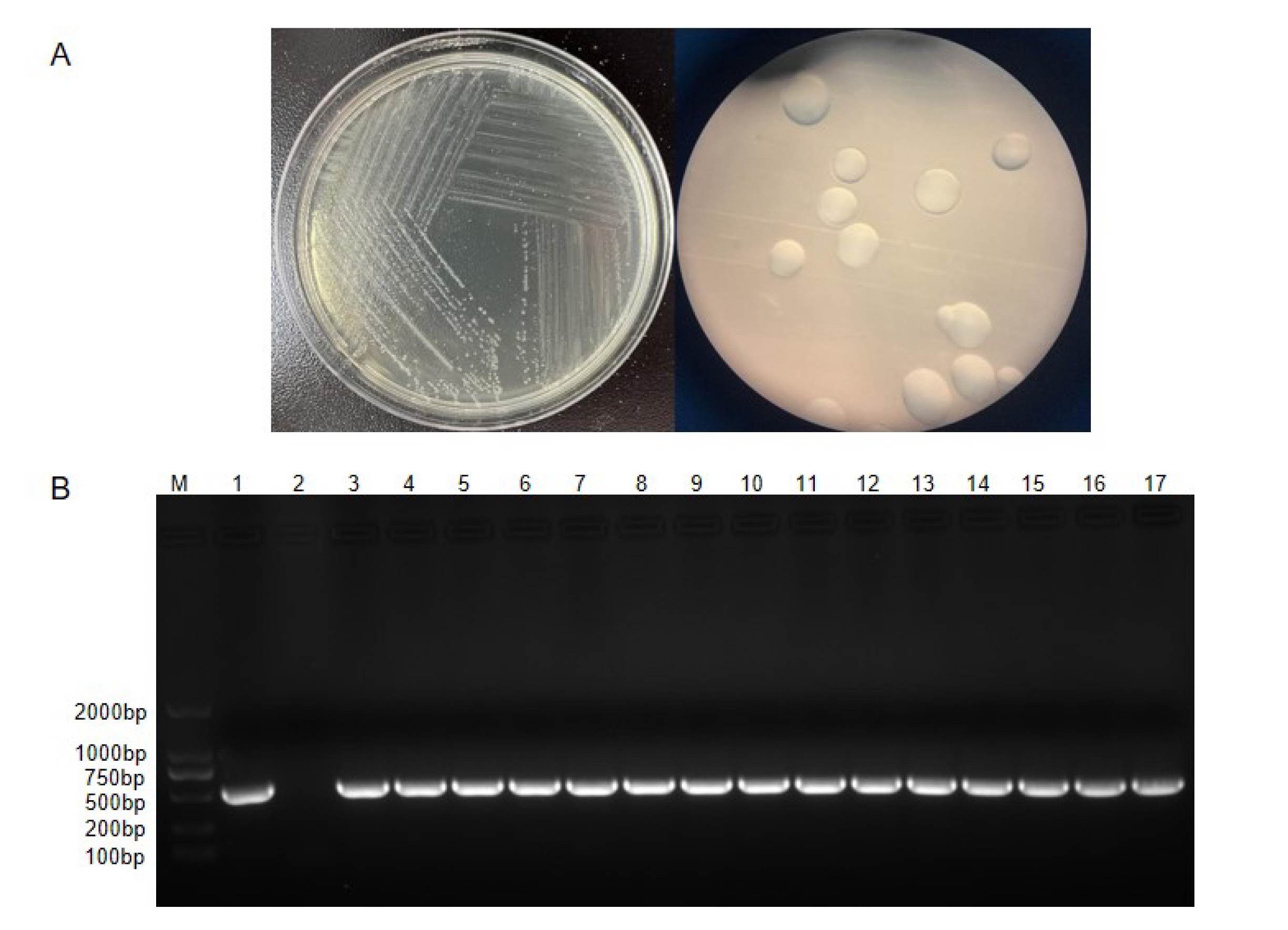

Supplement: Supplementary file 3 — Supplementary Material 3. [file 12866_2026_5122_MOESM3_ESM.jpg]

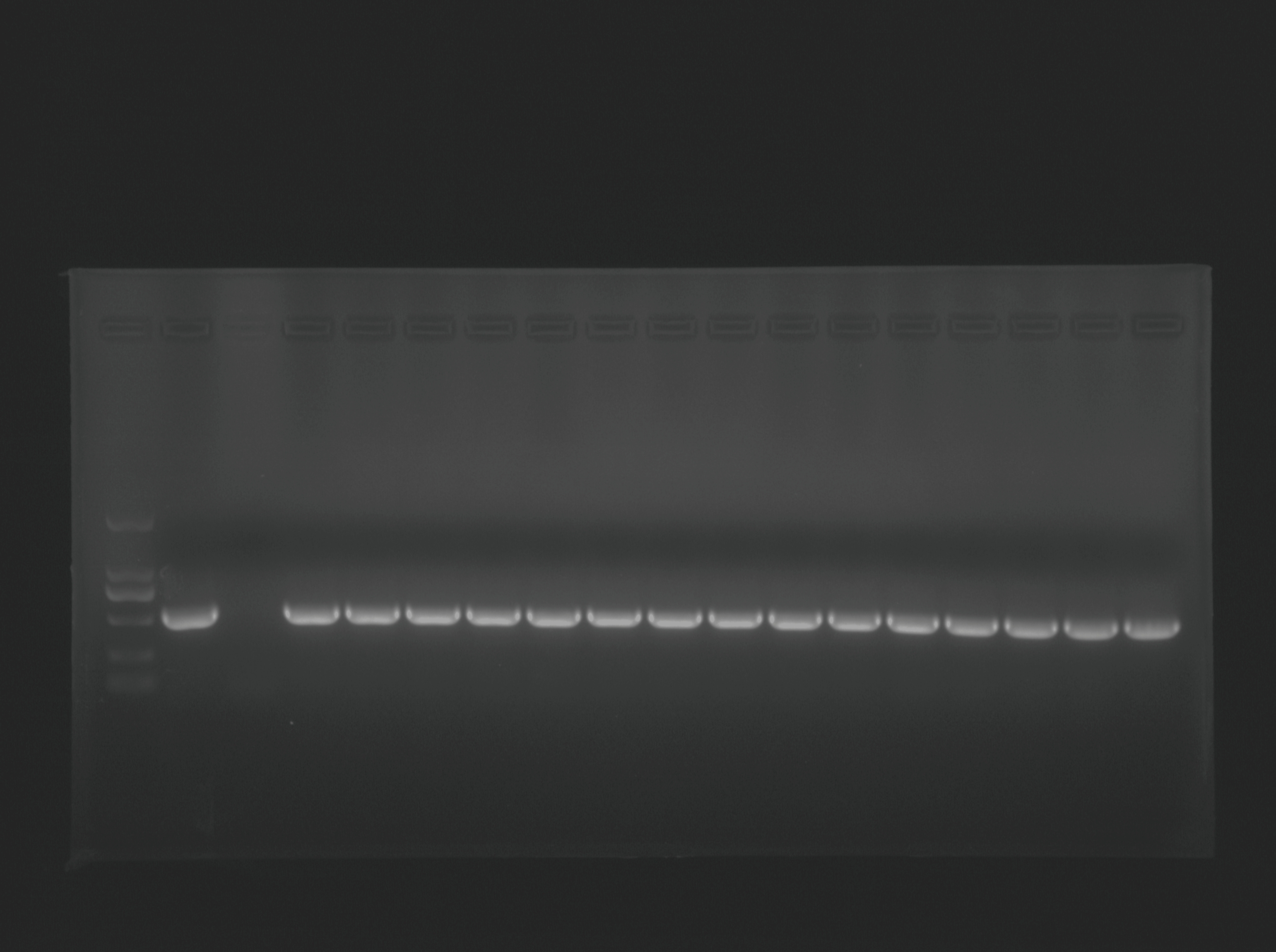

Supplement: Supplementary file 4 — Supplementary Material 4. [file 12866_2026_5122_MOESM4_ESM.jpg]
